# Standardized framework for evaluating costs of active case-finding programs: An analysis of two programs in Cambodia and Tajikistan

**DOI:** 10.1371/journal.pone.0228216

**Published:** 2020-01-27

**Authors:** Youngji Jo, Farangiz Mirzoeva, Monyrath Chry, Zhi Zhen Qin, Andrew Codlin, Oktam Bobokhojaev, Jacob Creswell, Hojoon Sohn

**Affiliations:** 1 Johns Hopkins Bloomberg School of Public Health, Baltimore, MD, United States of America; 2 Republican Centre of Population Protection from Tuberculosis, Dushanbe, Tajikistan; 3 Cambodia Anti-Tuberculosis Association, Phnom Penh, Cambodia; 4 Stop TB Partnership, Geneva, Switzerland; The University of Georgia, UNITED STATES

## Abstract

**Introduction:**

Over the years, technological and process innovations enabled active case finding (ACF) programs to expand their capacities and scope to have evolved to close gaps in missing TB patients globally. However, with increased ACF program’s operational complexity and a need for significant resource commitments, a comprehensive, transparent, and standardized approach in evaluating costs of ACF programs is needed to properly determine costs and value of ACF programs.

**Methods:**

Based on reviews of program activity and financial reports, multiple interviews with program managers of two TB REACH funded ACF programs deployed in Cambodia and Tajikistan, we first identified common program components, which formed the basis of the cost data collection, analysis, reporting framework. Within each program component and sub-activity group, cost data were collected and organized by relevant resource types (human resource, capital, recurrent, and overhead costs). Total shared, indirect and overhead costs were apportioned into each activity category based on direct human resource contribution (e.g. a number of staff and their relative level of effort dedicated to each program component). Capital assets were assessed specific to program components and were annualized based on their expected useful life and a 3% discount rate. All costs were assessed based on the service provider perspective and expressed in 2015 USD.

**Results:**

Over the two program years (April 2013 to December 2015), the Cambodia and Tajikistan ACF programs cumulated a total cost of $336,951 and $771,429 to screen 68,846 and 1,980,516 target population, bacteriologically test 4,589 and 19,764 presumptive TB, diagnose 731 and 2,246 TB patients in the respective programs. Recurrent costs were the largest cost components (54% and 34%) of the total costs for the respective programs and Xpert MTB/RIF (Xpert) testing incurred largest program component/activity cost for both programs. Cost per screening was $0.63 and $0.10 and cost per Xpert test was $25 and $18; Cost per TB case detected (Xpert) was $373 and $343 in Cambodia and Tajikistan.

**Conclusions:**

Results from two contextually and programmatically different multi-component ACF programs demonstrate that our tool is fully capable of comprehensively and transparently evaluating and comparing costs of various ACF programs.

## Introduction

The ambitious End TB Strategy aims for reduction in tuberculosis (TB) incidence and mortality rates to 90 and 95% of 2015 estimates by 2035; yet, current estimated annual rate of decline for TB incidence (2%) and mortality (3%) is far below the rate needed (10% or more) to achieve these targets.[1] With more than 3 million people with TB undetected contributing to the persistent transmission of the disease[2] intensified systematic screening programs for people with active TB, also known as active TB case finding (ACF), has a crucial role in halting disease transmission and improving care cascade for existing TB patients[3], ultimately leading to improving disease prevalence and incidence in high TB burden settings.[4]

In the past decade, multiple technological and process innovations enabled ACF programs to expand the capacity and scope beyond community symptom screening. Some examples of these innovations include involving mass media, educational programs, community engagements to increase awareness of the TB problem [5–7]; use of innovative technology such as Xpert MTB/RIF (Xpert) testing [8]and drones for sputum collection ensure timely diagnosis; Medication Event Monitoring Systems (MEMS) and mobile health (mHealth) tools to improve patient management [9–13]. Given the resource intensive nature of ACF programs, these additional innovations must be carefully evaluated for costs against their economic and operational feasibility and sustainability. However, the increasing complexity of ACF program design and operations pose considerable challenges in properly assessing the costs of these interventions, particularly in evaluating cost drivers and by key program components.

While there is growing evidence demonstrating the public health impact of ACF programs, evidence on costs of ACF programs is far too scarce [14, 15]. This is largely attributable to de-prioritization and technical challenges in conducting proper costing studies alongside complex program operations without expertise or standardized tools. Existing costing studies in TB are challenged with a lack of consistency and transparency in their methodologies and reporting [16], making it difficult to properly value these important programs in terms of their cost-effectiveness, affordability, and sustainability. Likewise, there is a need for a generalizable costing tool for ACF programs that is adherent to the guidelines [17], standardized in reporting estimates.

Therefore, the overall aim of our study was to develop a generalizable and comprehensive cost data collection, analysis, and results reporting framework that can be adopted for retrospective cost assessment of a wide range of ACF programs. The use of such a tool can facilitate systematic and transparent comparison of costs–total program costs, service (e.g. screening, diagnosis, and treatment) unit, and program yield costs–of ACF programs. To demonstrate the data categorization, analysis, and reporting process using this framework, we report costs of two operationally different multi-component ACF programs targeting different geographical areas and populations that operated in Cambodia and Tajikistan supported by the TB REACH (Stop TB Partnership, UNOPS) wave 3 funding cycle.

## Methods

### Description of TB REACH active case finding programs

The TB REACH initiative of the Stop TB Partnership (UNOPS) is a multi-lateral funding mechanism supported by the Global Affairs Canada and the Bill and Melinda Gates Foundation with specific aims to improve TB case detection and care cascade in high burden countries by funding innovative projects. During the wave 3 funding cycle, TB REACH funded a total of 32 projects across 24 countries. In this study, we used two TB REACH wave 3 funded programs in Cambodia and Tajikistan to demonstrate the development, data collection and analysis processes of our costing framework. These two programs were one of the more complex, multi-component programs that targeted widely different geographic areas (rural vs. urban), population and health systems (patients making outpatient visits to public health clinics vs. community-based screening of the elderly), and epidemiological settings (Cambodia and Tajikistan rank one of the top 30 highest burden countries for TB incidence and MDR-TB at an incidence of 302 per 100,000 and 20 per 100,000 respectively as reported in 2019).[1]

In Cambodia, TB REACH funded the Cambodia Anti-Tuberculosis Association (CATA), a non-government organization (NGO), working in partnership with the national tuberculosis program (NTP) to implement an ACF program focused on elderly (aged 55 years or older) and other vulnerable groups in the remote, rural areas.[18] This program covered 194 health centers (HC) in 13 operational districts (OD) in Cambodia between April 2013 to May 2015, screening a total of 125,842 patients. At each HC, CATA-trained the Village Health Support Groups (VHSGs) conducted a one-week long community house-to-house symptom screening and presumptive TB patients identified were invited to attend the one-day mobile diagnosis service (using a vehicle equipped with mobile X-ray and GeneXpert IV units) hosted at respective local HC. Patients were initially screened with Chest X-ray (CXR) and those with abnormal CXR and suspected of drug-resistant TB were bacteriologically tested using Xpert assay. Patients with positive Xpert test and CXR active for TB (clinical evaluation) were referred to the HC for treatment initiation.

In Tajikistan, TB REACH funded the NTP to implement an ACF program covering 56 policlinics, 1 pre-detention, and 1 diabetes center. Between April 2013 and December 2015, the Tajikistan ACF program–consisted of symptom screening using a questionnaire-based tool installed on a mobile phone device, followed by CXR and bacteriologic testing using AFB smear (routine) and Xpert (part of the ACF program) based on the symptom screening results, referral for treatment initiation–screened a total of 849,215 patients visiting policlinics, 22,078 in a detention, and 1,066 at a diabetes center.

### Cost analysis framework and data collection

**Fig 1:**
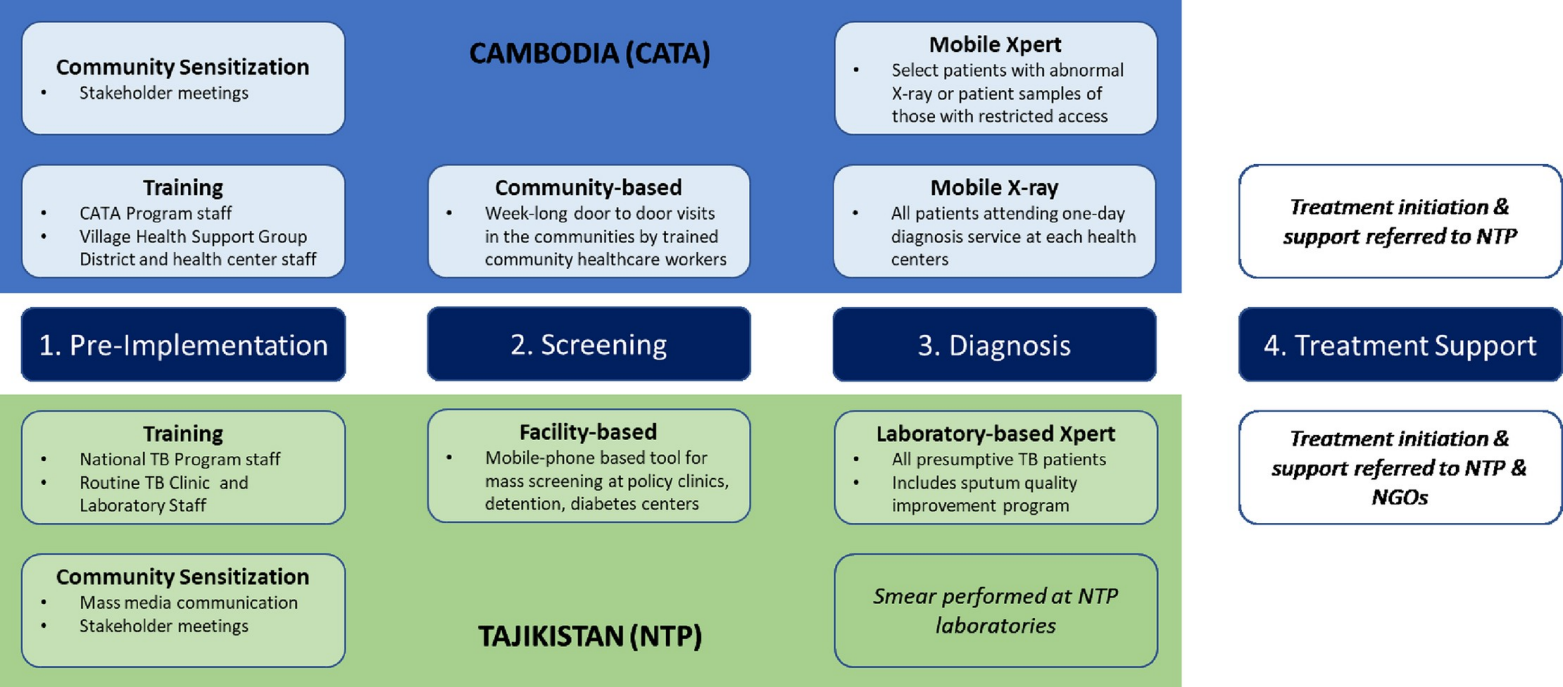
Conceptual framework of operation components of the active case finding programs in Cambodia and Tajikistan.

As our primary aim was to develop a standardized activity-based cost data collection tool and analytic framework [19, 20] that can be used for retrospective evaluation of various multi-dimensional ACF programs, we first defined 4 major activity categories– 1. Pre-implementation activities, 2. Patient screening, 3. Diagnosis, and 4. Treatment provision–common across all 32 TB REACH wave 3 projects (Fig 1). Within each major activity category, we further declassified unique activities based on major activities identified in our detailed review of the Cambodia and Tajikistan program (see S1 File). For example, under “Pre-implementation activities”, we further distinguished resources mobilized for two distinct types of activities (e.g. “community sensitization meetings vs. program training”) that were performed prior to ACF program operated in the target settings (e.g. clinics, facilities, and/or communities). Within screening, diagnostic and treatment categories, we allowed for different permutations of screening, diagnosis and treatment services offered by ACF programs that may depend on types of technologies (e.g. mobile-health tools for screening, mobile Chest X-rays, Xpert MTB/RIF assay) used, duration and location of the program operations. [21]

To allow for evaluation of key cost drivers, we designed the data collection tool to categorize resources into four distinct categories (human resource, capital assets, recurrent goods, and overhead costs), where individual cost items can be identified and allocated by relevant activity components in a matrix format (Table 1 and S1 File). Human resource costs for each activity component were estimated based on their estimated level of effort (LOE), approximated as proportional time allocation (%) of their full-time work spent on each activity during the retrospective one year of program operations. For the costs of goods, equipment, and services, we divided direct costs into capital and recurrent costs based on the ‘purpose’ of the activities and items (instead of simply dividing them by an expenditure cycle). For example, we considered not only capital assets but also program administrative and operational (overhead) costs (e.g. rent, staff per diem, travel costs, refreshments, meeting/communication materials, office rent) during the ‘pre-implementation phases’ (e.g. community sensitization and training) as capital costs, since these can be considered one-time fixed costs as the two programs are implemented in a given catchment area (without increasing number of staff, geographical coverage or expanding facilities) and do not recur through program implementation and are not likely varying by program outputs such as numbers of patients served in a given catchment area. (i.e. If additional training or community sensitization are needed with geographical expansion during the program implementation, these costs will be considered as recurrent costs.) Recurrent costs included direct resources mobilized for carrying out program implementation of screening, diagnosis, and treatment—that changes in proportion with program output (here, the number of patients served). Total shared, indirect and overhead costs were first calculated and then apportioned into each activity category based on direct human resource contribution–average respective LOE (%) based on a number of staff and their relative time for each activity category–to reflect the resource utilization level of each activity group. Overhead costs and capital assets (e.g. mobile van, GeneXpert systems) were both assessed as an annual cost based on the total duration of the project operation (2 years) or duration of their useful life (between 2 and 15 years) and a 3% discount rate (WHO-CHOICE).[17] Details on the overall process of data collection, allocation, and source of the data are provided in S2 File and S1 Table of the supplement document.

**Table 1:** Analytic and reporting framework for total, activity-specific, and program yield costs of active-case finding programs.

**Cambodia (CATA)**		**Pre-implementation**			**Screening**	**Diagnosis**		**Treatment**
Resource categories	Total cost (%)	Community Sensitization	Ad-hoc staff training	Staff training	Community based	CXR	Xpert	Treatment
Human resources	$57,728 (17%)	$32,277	$3,791	$477	$18,212	$1,485	$1,485	NA
Capital cost	$71,964 (21%)	$2,003	$5,787	$1,016	$119	$34,468	$28,571	NA
Recurrent costs	$183,018 (54%)	$0	$0	$0	$53,232	$43,136	$86,650	NA
Overhead costs	$24,241 (7%)	$13,554	$1,592	$200	$7,647	$624	$624	NA
**Total activity costs**	**$336,951**	**$47,834**	**$11,171**	**$1,693**	**$79,210**	**$79,712**	**$117,330**	NA
**Incremental costs for yield**					**$139,908**^**3**^	**$204,198**^**4**^	**$272,660**^**5**^	NA
Types of services	Number of beneficiaries	Cost per activity^1^						
Population enrollment	125,842	$0.38	$0.09	$0.01	$0.63			
CXR test	23,797					$3.35		
Xpert test	4,604						$25.48	
Treatment referred	4,604							NA
Program yield indicators	Number of yield				Cost per yield^2^			
Presumptive TB cases identified	28,038				$4.99			
TB cases diagnosed by all form TB (including CXR or routine clinical evaluation)	1,427					$143.10^6^		
TB cases diagnosed by Xpert	731						$373	
Treatment completed	NA							NA
**Tajikistan (NTP)**		**Pre-implementation**			**Screening**	**Diagnosis**		**Treatment**
Resource categories	Total cost (%)	Community Sensitization	Ad-hoc staff training	Staff training	Facility based	CXR	X-pert	Treatment
Human resources	$157,509 (20%)	$43,253	$2,591	$14,859	$93,866	NA	$2,941	NA
Capital cost	$190,614 (25%)	$12,525	$4,453	$17,674	$93,336	NA	$62,625	NA
Recurrent costs	$258,792 (34%)	$0	$0	$0	$3,310	NA	$255,482	NA
Overhead costs	$164,513 (21%)	$42,042	$40,214	$42,042	$10,968	NA	$29,247	NA
**Total activity costs**	**$771,429**	**$97,820**	**$47,258**	**$74,576**	**$201,480**	NA	**$350,295**	NA
**Incremental costs for yield**					**$421,134**	NA	**$771,429**^**7**^	NA
Types of services	Number of beneficiaries	Cost per activity						
Population enrollment	1,980,516	$0.05	$0.02	$0.04	$0.10			
CXR test	NA					NA		
Xpert test	19,764						$17.72	
Treatment referred	19,764							NA
Program yield indicators	Number of yield				Cost per yield			
Presumptive TB cases identified	22,086				$19.07			
TB cases diagnosed by all form TB^8^	3,979					NA^8^		
TB cases diagnosed by Xpert	2,246						$343.47	
Treatment completed	NA							NA

1. Cost per activity is calculated by respective activity costs divided by a number of relevant beneficiaries served.

2. Cost per yield is then calculated by respective incremental screening/diagnostic test costs divided by numbers of relevant service outputs such as presumptive TB cases determined by screening and TB cases diagnosed by Xpert/CXR.

3. The total Xpert cost also include costs incurred by the routine clinic service (i.e. clinical evaluation + smear performed) before Xper test.$139,908 = $47,834 + $11,171 + $1,693 + $79,210; we incrementally added the relevant activity costs–upto screening—for presumptive TB identified as those (27,554) are the result of the pre-implementation and screening activities.

4. $204,198 = $47,834 + $11,171 + $1,693 + $79,210 + $79,712 - (4,604*$3.35); we incrementally added the relevant activity costs–upto CXR—for TB cases diagnosed by CXR (1,425) but deducted (4,604*$3.35) from the total CXR activity costs ($79,712) because 4,604 people further required Xpert test to determine TB except extrapulmonary tuberculosis.

5. $272,660 = $47,834 + $11,171 + $1,693 + $79,210 + (4,604*$3.35) + $117,330; we incrementally added the relevant activity costs–upto X-pert except CXR–for TB cases diagnosed by X pert (728). We only added (4,604*$3.35) out of $79,712 from CXR cost in order to account only attributed CXR costs for those (4,604 out of 23,797) who also received X-pert tests after abnormal CXR.

6. This cost per yield ($143.10 in Cambodia) is related to referred patients by the CATA program for all non X-pert testing patients to the routine clinic as it operated at the health center. Considering the full diagnostic algorithms including routine clinic evaluation and bacteriologic testing, the costs per yield may be greater than our current estimates, we did not include these costs as they were outside the scope of the ACF operations.

7. In Tajikistan, we incrementally added activity costs for TB cases diagnosed by Xpert. Likely, the incremental costs for yield may vary depending on the diagnostic algorithms. To calculate an accurate cost per TB cases detected, it is therefore important to carefully account attributed costs to the particular yield–not simply using a total program or activity costs—based on patients' flows.

8. This figure includes TB patients independently identified by the National TB Program; thus, we denoted “NA” for the cost per program yield cell of the reporting table as we are not able to further declassify relevant resource used for those TB patients identified outside and costs for those yield estimates that are not solely or activity category cells which were not included into our costing analyses.

The main outcomes of our costing framework are total costs, service unit costs, and cost per program yield. Total costs are tallied by each programmatic component as well as key resource type as shown in Table 1. Service unit cost (i.e. cost per activity) is calculated based on the total programmatic costs and respective beneficiaries for each service component of the ACF program (e.g. cost per Xpert test). Cost per program yield was assessed based on to the sum of the costs of all relevant programmatic components (e.g. cost per TB patient diagnosed is calculated based on the sum of total programmatic costs for pre-implementation, screening, and relevant diagnostic services and the total number of TB patients diagnosed) then calculated by respective program yield estimates for presumptive TB patient screening or TB diagnosis (e.g. bacteriologic diagnosis via Xpert test).

All costs were assessed from the ACF program perspective and did not include costs associated beyond the program operations (e.g. ‘routine’ TB program costs such as routine diagnostic or treatment managed by NTP or clinic). All costs were evaluated as economic costs from the program perspective, presented in 2015 US dollars with relevant adjustment for inflation using the International Monetary Fund (IMF) consumer price indices where necessary.[22] We used multiple data sources (TB REACH wave 3 application and reports, budget and financial reports, and iterative discussions with program managers) of the respective programs to identify all types of direct and indirect costs associated with each program operation and performance. Furthermore, in order to fully account for the opportunity costs, we further designed the data collection tool to capture data on donated goods, volunteer efforts (e.g. government staff, local temporary hires/volunteers recruited and trained as part of the ACF program) [23], and other cost items that were not included in the financial statements, but identified via additional interviews with the program managers and review of program reports.

### Ethics

Our study did not involve human participants nor utilize patient data. As such, institutional review board/ethics approval was not required.

## Results

Following our conceptual framework (Fig 1), we developed a standardized reporting table for all relevant cost estimates (Table 1). We designed this table to report the total program costs stratified by key activity components and resource categories that can be directly compared across ACF programs, along with program service outputs (numbers of patients screened, receiving diagnostics services, etc.) and yield (numbers of presumptive TB patients identified and TB patients diagnosed by respective diagnostic services).

**Fig 2:**
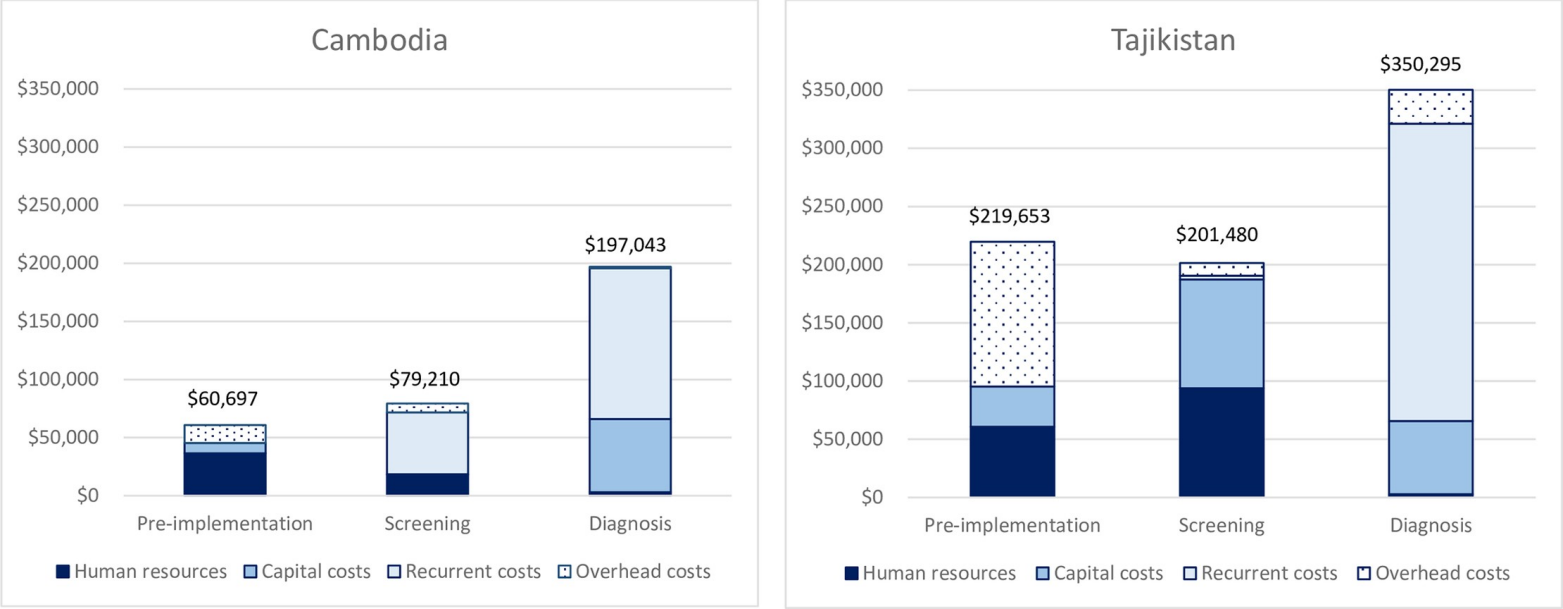
Total program costs and relevant cost drivers of the multi-component active case finding programs in Cambodia and Tajikistan.

Based on the analytic and reporting framework, we report that the total costs of the respective ACF programs were $336,951 and $772,429 for Cambodia and Tajikistan, spread over two program years. From the resource use perspective, the major cost drivers were recurrent costs such as Xpert cartridge, per diems to the field team, and vehicle rental and fuel (54%), followed by capital costs (21%) and human resources (17%) in the Cambodia program. Similarly, in the Tajikistan program, recurrent cost mainly Xpert cartridge (34%) was the most significant cost driver, followed by capital cost (25%), overhead (21%) and human resource cost (20%). Based on the six key activities components, both programs showed similar trends with the majority of costs were associated with the diagnosis (59% vs. 45%), followed by screening (24% vs. 26%) and community sensitization (13% vs 14%) for Cambodia and Tajikistan respectively, except Tajikistan had higher cost allocation for training (16% vs 4%) than the Cambodia program. A summary of cost distribution by resource type for each program is illustrated in Fig 2.

Program activity costs were highly dependent on the scope of the screening strategies and program’s target populations. The Tajikistan program screened nearly 30-times the number of patients (1,980,516 patients) compared to the Cambodia program (125,842 patients) over the span of two program years. Subsequently, cost per patient screened was approximately 6 times higher at $0.63 for Cambodia’s program compared to $0.10 in the Tajikistan program. However, the Cambodia program had a much higher rate of identifying presumptive TB patients with 5 patients needed to be screened to identify 1 presumptive TB patient (125,842 patients screened / 27,557 presumptive TB patients identified) compared to 90 patients needed to be screened in the Tajikistan program (1,980,516 / 22,086). On the other hand, cost per for Xpert test differed between Cambodia and Tajikistan at $25 vs. $18, largely attributable to the differences in utilization level of capital assets (e.g. GeneXpert machine) and overhead costs. While the facility-based Xpert testing in Tajikistan had much larger total program costs compared to the community based operation in Cambodia, lower testing volume due to selective Xpert testing (based on CXR results) resulted in a less utilization rate (49%) in the Cambodia program compared to 57% in the Tajikistan program (given 16 tests per day per one GX4 unit) of the capital assets and program overheads.

Subsequently, program yield costs were much higher for the Tajikistan program at $19 per presumptive TB patient identified compared to $5 for Cambodia’s program. Overall cost per TB patient identified (all combined diagnosis: bacteriologic with smear and/or Xpert and clinical) through respective programs were $343 in Tajikistan and $156 (336,951 total program costs / 2,158 TB cases confirmed by all form TB and Xpert) in Cambodia. Focusing on our comparisons of common diagnosis platform (Xpert) between the two programs, the Cambodia’s program had slightly higher yield in diagnosing TB patients using Xpert (16%, 728/4,604) compared to Tajikistan (11%, 2,246/19,764), but costed slightly more to diagnose one TB patient ($373 vs. $343).

## Discussion

In this study, we developed a comprehensive and generalizable framework for cost data collection, analysis, and reporting that can be used to evaluate and compare costs of various ACF programs deployed in a wide range of settings and target populations. In developing this framework, we focused on the operational aspects of the ACF programs and reviewed multiple data sources from the two different multi-component ACF programs in Cambodia and Tajikistan, to define common activity components within which resources consumed can be summarized in a transparent and comparative manner. Using this framework, we were able to apply the same methodologic standards in collecting and analyzing data, and reporting costs of respective ACF programs. Furthermore, using our reporting framework, we were able to identify differences in the major cost drivers (Xpert operation based on the activity categories, contributing to 35% vs. 45% of the total program costs for Cambodia and Tajikistan) and factors contributing (operational algorithms and diagnostic criteria resulting in differences in service volumes) to differences in service unit and program yield costs between the two programs.

Service unit costs for Xpert and program yield cost for identifying one presumptive and confirmed TB patient were largely different between the two programs due to the patient volumes subjected to each service components of the program and differences in the operational algorithms (e.g. selective Xpert testing for those with abnormal CXR in Cambodia vs. non-selective upfront Xpert testing for Tajikistan). While the larger scope of program operations in Tajikistan resulted in higher total costs for each program components and greater number of patients screened and subjected to bacteriologic testing, service unit cost for Xpert was much higher in the Cambodia program at $25 (vs. $18) due to its selective diagnostic algorithm for Xpert, reducing overall testing volume and the level of testing capacity utilization. On the contrary, costs of program yields depended on the effectiveness of the strategy in identifying presumptive and active TB patients, resulting in fairly similar estimates ($373 and $343) in both countries. While the GDP per capita in Tajikistan ($3,015) is almost three times than Cambodia ($1,384), the lower unit cost per service and cost per yields suggest the TB REACH program in Tajikistan is likely more economically efficient and affordable compared to the TB REACH program in Cambodia.

To assess validity of our unit cost estimates, we compared our results with previously published estimates of TB diagnostics and interventions. [16, 24–27] Focusing on cost per Xpert test, we find our estimates consistent and comparable to the existing evidence (between $12 and $42 per test) factoring operational settings, costing methods, consumables costs, and testing volumes. In comparing our cost per program yield estimates, we found one study that reported costs of program operation and yields of the CATA program that overleaped with our assessment period but reporting much lower cost estimate. [14] We noted several important methodologic differences between our study and the study by James et al [13] that may have attributed to this difference. First, James et al., evaluated only the first 12 months of the program that were funded by TB REACH wave 3 whereas we evaluated CATA’s two full program years under the same funding scheme. Second, comparing the program’s first year cost data, we noticed that James et al. study did not fully account for costs incurred by the program beyond the financial statements. For example, James et al., did not include costs of equipment, donated goods, or local volunteers dedicating their time for training and fieldwork for the CATA program. Furthermore, while the CATA program had two distinct diagnostic algorithms (post CXR screening referral to routine clinic vs. on-site mobile Xpert testing), James et al., did not report these important sub-programmatic services and yield estimates that made it difficult to directly compare sources of differences in our cost estimates.

The use of our costing framework has several advantages over other costing tools available TB services [28][29]. First, our tool is focused on evaluating costs of ACF programs. This allows for a much simplified and unified approach in data collection, analysis, and standardized reporting of results that are categorized based on common activity components across a wide range of ACF programs (at least those programs similar to the TB REACH wave 3 projects). Likewise, our two program examples demonstrate 1) the versatility of our tool in systematically collect, analyze, and report key cost estimates (total costs, service unit costs, and cost per program yield) of two very different programs, 2) ability to compare key cost drivers of the respective programs, and 3) to generate service unit costs that may be useful in informing model-based cost-effectiveness analyses. Second, while our two program example only use full programmatic costs (i.e. costs incurred over the entire program period), our costing framework can be extended to tally and analyze costs on a quarterly basis (or in much less or longer time steps, depending on how frequent the end-users of the tool are able to work with program managers and finance officers to collate and declassify costs and program yield estimates). This would allow for an assessment of cost trends against various program implementation and operational factors (service volume, program output and changes in service coverage) and further inform how programs’ ‘economies (or diseconomies) of scale’ are achieved and influenced by these factors.

## Limitations

Results from our study should be interpreted with the following notion of limitations. First, we were only able to evaluate program costs and activity outputs retrospectively, limiting our analytic option to the top-down method. Therefore, we were not able to capture uncertainties in cost estimates that may arise due to variabilities in operations and workloads that may depend on operational settings conditions.[24, 26, 30] This limitation can be overcome by designing costing studies alongside the program development and implementation to include staff time-use surveys or time and motion and tracking operational statistics so that these data can help inform cost allocation mechanisms, describe uncertainties in cost estimates associated with operational variabilities and activity-specific inefficiencies [24, 31]. Subsequently, we recommend that costs be routinely evaluated so that variability in operations and settings can be captured and reflected as part of the cost reports. Second, as with James et al. study, it is important to note that our program service (e.g. per-test cost) and yield cost estimates (e.g. cost per TB case detected) does not fully capture population characteristics, TB epidemiology in the program’s catchment area, and operation management of the program that will likely influence the program’s performance. Likewise, additional studies evaluating periodic workloads, program population coverage, and overall cost-effectiveness of the program against comparators (e.g. status quo, or other types of TB control programs) can supplement overall performance, utility, feasibility, and sustainability of the ACF program beyond current operations.

## Conclusions

Our evaluation of costs of two contextually and programmatically different ACF programs in Cambodia and Tajikistan demonstrate that our comprehensive costing data collection, analysis, and reporting framework can be used to evaluate and compare costs of a wide range of ACF programs in a highly transparent manner. Furthermore, in efforts to standardize costing practices, a similar approach can be adapted to evaluate other TB control programs (and possibly other vertical programs such as malaria or HIV/AIDS) and services. However, retrospective top-down assessment of costs restricts the scope of how costs can be assessed and do not allow evaluation or monitoring of factors that influence the costs of operations. More research is needed in this regard, early and periodic (e.g. quarterly or monthly) assessment of costs using the proposed framework is highly encouraged.

## Supporting information

S1 File Principles and definitions of activity-based costing categories. (DOCX)

S2 File Process of data collection and communication with the program personnel. (DOCX)

S1 Table Cost data elements for cost analysis of TB active case finding program and suggested data sources. (DOCX)
