# Supplementary material for: Standardized framework for evaluating costs of active case-finding programs: An analysis of two programs in Cambodia and Tajikistan
Source: PLoS One. 2020 Jan 27;15(1):e0228216. doi: 10.1371/journal.pone.0228216 (PMC6984737; doi:10.1371/journal.pone.0228216)
Supplement: S1 File — (DOCX) [file pone.0228216.s001.docx]

**S1 File.** **Principles and definitions of activity-based costing categories**

- 1. **Activity categories**

**Community sensitization:** Activities aim to mobilize local leadership, guild consensus and raise public awareness of the program in the community. Activities include community mapping, local government stakeholder meetings, or mass campaigns in the villages.

**Training:** Activities aim to train ad hoc staff/on-demand volunteers/government staff who are hired or recruited temporarily (Ad hoc staff training) and to train regular program staff for overall program implementation (program staff training). Activities include training for screening (active case finding, house to house visits, patient enrollment etc.), the conduct of diagnostic practice (CXR and X-pert) and treatment component (Directly Observed Therapy, DOT monitoring) of the TB REACH program.

**Screening:** Activities aim to register the target population and screen TB suspects. Activities include population enrollment and conduct systematic symptom screening to detect TB suspects. Screening may be conducted in the community settings through the door to door visits of households of TB suspects or risk groups (active case finding) or at the facility settings through passive surveillance. Screening tools may use a mobile phone or tablet-based platforms.

**Diagnosis:** Activities aim to test TB suspects and diagnose TB patients. A diagnostic algorithm may have various permutations of diagnostic tests including routine clinic service (i.e. clinical evaluation and smear performed), X-ray, or X-pert or other lab tests. Patients who were diagnosed as TB cases were referred to public clinics for treatment.

**Treatment:** Activities aim to refer TB patients for the initiation and continuation of TB treatment. Activities include outpatient care or hospitalization based on the severity of TB conditions as well as drug adherence strategies such as DOT monitoring.

- 1. **Resource categories**

**Human resource** includes not only program staff members but also any ad-hoc staff, government staff or staff in other organizations who were, directly and indirectly, involved in the program implementation. We collected monthly salaries, duration of employment and estimated proportional time allocation (expressed as FTE/LOE over retrospective one year time horizon) to each activity component of the ACF program. For volunteers or the personnel without salary information, we matched their estimated salaries based on the level of expertise and types of tasks performed with inputs from the program managers.

**Capital cost** is a fixed, one-time costs associated with a project, which is constant to production (here, project operation) output. The cost may include not only capital assets but also activity costs and office maintenance costs (e.g. rent, staff per diem, travel costs, refreshments, meeting/communication materials, office rent), during the preparation phases -- community sensitization and staff meeting --as capital costs, since these can be considered one-time costs for the entire program and do not recur through program implementation.

**Recurrent cost** is a cost of labor, material or overhead that changes in proportion with production output. These costs include direct resources mobilized for carrying out program implementation of screening, diagnosis, and treatment. Recurrent costs included in this category included staff per diem (in addition to regular staff salaries), travel costs, capital assets (e.g. vehicles, GX4, mobile X-ray), medical and general consumables as well as patient incentives, if any.

**Overhead cost** includes office rent, general office supplies/maintenance costs, other general equipment or furniture (e.g. computers, non-ACF program specific vehicles or desks, etc.) or on-going programmatic supervision expenses that are classified as indirect costs to program operations (fuel & gas, water, electricity, phone/internet, biosafety waste management etc). We excluded monitoring and evaluation (M&E) costs in this cost analysis since our costing analyses focus on program implementation so the results can be systematically compared with other programs in different settings.
