# Supplementary material for: Standardized framework for evaluating costs of active case-finding programs: An analysis of two programs in Cambodia and Tajikistan
Source: PLoS One. 2020 Jan 27;15(1):e0228216. doi: 10.1371/journal.pone.0228216 (PMC6984737; doi:10.1371/journal.pone.0228216)
Supplement: S2 File — (DOCX) [file pone.0228216.s002.docx]

**S2 File. Process of data collection and communication with the program personnel**

- We first share the tool with the program manager to identify a specific time period, operational stages, screening and diagnostic algorithms, and activity subcomponents into relevant activity groups. Through the retrospective data collection process, we took an ingredient approach-- where the number of inputs was multiplied by prices of each respective item. We identify and fill the relevant budget/expenditure line items (unit costs and quantities) into resource input categories based on budget/expenditure/administrative data and interview with program managers.
- We then calculate resource costs by combining relevant unit costs and quantities (e.g. number of items, working months) as well as a percentage of utilization in case of any shared resources. The total direct TB diagnosis and treatment costs (medicines, tests, treatment monitoring costs, etc.) are multiplied by the number of TB cases treated to arrive at a total direct cost. For example, when the quantity data of cost inputs are not available (e.g. total cost of Xpert cartridge), we used other relevant measures to estimate the quantity such as number of patients who received service (e.g. GeneXpert test) or number of events (e.g. number of mobile clinic operations) based on generally expected frequency in a given time period. Where there are any donated or shared items from other programs, we identify local market prices and the approximate percentage of utilization based on available references such as a number of tests/patient volume.
- All costs—financial expenditure, staff time, procured equipment, supplies and resources—attributable to their underlying activities were identified and carefully allocated directly and proportionally (based on FTP/LOE) to relevant activity category. In this way, under the general categories of activity grouping, the various resource cost ingredients are allocated to the relevant implementation stages and operational activities which allows better visibility in identifying cost drivers from program management perspectives.
- For any shared resource costs across activity groups (such as human resource and overhead costs), we identify proportional resource utilization estimates to each activity group based on time spent or operational volume.

1. Human resource costs can be initially calculated based on number of staff, number of working months and amount of monthly salaries. When the staff involve in multiple programs, we initially identify general time allocation to the TB REACH program (e.g. 50%). We then allocate the 50% of the total cost into relevant activity group based on level of effort (%) estimates. The estimates can be determined by 1) time motion study/daily report form (prospective data collection), 2) self-reported monthly/quarterly level of effort by staff, and 3) approximate percentage of contribution to each activity by the end of the program (retrospective data collection).
2. Overhead cost can be proportionally allocated to activity groups based on relevant proxy estimates of resource utilization. There are several criteria used in allocating overhead costs in a cost analysis such as size of the staff (number) or human resource cost, size of the building space, value of capital asset or budget allocated, or volume of tests/services output. The choice of overhead allocation method should be decided based the key factors that generate overhead costs in the long-run. In this macro costing analysis, we apportioned the overhead costs by the size of the staff (number) or human resource cost since we thought staff hands-on time is a good proxy for utilization of the overhead costs during the program implementation. When there is no information available for overhead costs, some study may use any flat rate (20%) which is apportioned equally across all activity groups.

- We make necessary adjustments for capital costs (annualization, inflation/discounting) and recurrent costs (number of tests, other increasing quantity over time) to calculate total resource and activity costs.
- We identify a number of beneficiaries served by each activity (training, screening, diagnostic tests, treatment etc) by monthly/quarterly, if possible, or by the end of the program implementation.
- We calculate cost per activity (screening/test) and cost per yield based on the number of beneficiaries. Cost per activity is calculated by respective activity costs divided by number of relevant beneficiaries served. Cost per yield is then calculated by respective incremental screening/diagnostic test costs divided by numbers of relevant service outputs such as TB suspects determined by screening and TB cases diagnosed by X-pert/CXR.
