# Supplementary material for: Standardized framework for evaluating costs of active case-finding programs: An analysis of two programs in Cambodia and Tajikistan
Source: PLoS One. 2020 Jan 27;15(1):e0228216. doi: 10.1371/journal.pone.0228216 (PMC6984737; doi:10.1371/journal.pone.0228216)
Supplement: S1 Table — (DOCX) [file pone.0228216.s003.docx]

**S1 Table. Cost data elements for cost analysis of TB active case finding program and suggested data sources**

| **Key resource cost items, ingredients, and suggested data sources** | | | |
| --- | --- | --- | --- |
| **Resource categories** | **Cost items** | **Cost ingredients** | **Suggested source of data** |
| **Human Resources** | Program staff | Monthly salaries/level of effort/number of months of Project manager, coordinator, accounter, lab technician, X-ray technician, field supervisors, drivers, etc. | Government salary scale/hospital accounts office |
|  | Government staff | Monthly salaries/level of effort/number of months of nurses, lab technicians, doctors | Government salary scale/hospital accounts office |
|  | Ad-hoc staff | Per diem/monthly salaries of volunteers, other NGO workers | Program budget records/interview |
| **Capital cost** | Building | Building/clinics/infrastructure construction costs | Government Estates and building planning office / Construction contractors |
|  | Equipment purchases | X-ray machine, Gene X-pert machine, Vehicle, Mobile phones, Bicycles, Laptops, Smear microscopy, | Program budget/expenditure records |
|  | Community sensitization | Refreshers, space rental fee, number of trainers, per-diem for trainers/participants, printing, other advocacy activities, traveling costs | Program budget/expenditure records |
|  | Staff training | Refreshers, space rental fee, number of trainers, per-diem for trainers/participants, printing, other advocacy activities, traveling costs | Program budget/expenditure records |
|  | Other research & development | New screening tool development, operational research, monitoring, and evaluation costs etc | Program budget/expenditure records |
| **Recurrent cost** | Screening | Per diem for volunteers, incentives, patient cards, | Program budget/expenditure records |
|  | Diagnosis | All types of chemicals and reagents utilized for diagnostic methods evaluated / drugs used for TB specific and non-specific clinical practice (e.g. GeneXpert cartridges)  All types of general laboratory and medical consumables (e.g. latex gloves, syringe, etc.)  Cost of a vehicle used for specimen transport, List of locations referring specimens to the laboratory | Maintenance contracts for all laboratory equipment requiring periodic maintenance (Laboratory financial records/ service contractors/ manufacturer catalog (must include all costs associated with procurement, usually at 25% of the catalog price) |
|  | Treatment | Drug costs (1^st^ line/2^nd^ line treatment, MDR/XDR treatment)  Directly observed therapy (DOT), other incentives for referral,  External Quality Assurance/Control (QA/QC) for various laboratory diagnostic & clinical activities | Pricing/cost available through laboratory/hospital accounts office. Standard Operating Procedure (SOP) |
|  | Contact investigation | per diem/level of effort/number of volunteers  salaries/level of effort/number of field supervisors | Program budget/expenditure records |
| **Overhead cost** | Office supplies | desks, chairs, printers, laptop computer, camera, boxes, cabinet etc | Program budget/expenditure records |
|  | Office maintenance | Fuel/Gas, water, phone/internet fees, electricity, cleaning service, shipping, |  |
|  | Transportation costs | Gas, fuel, car battery, Insurance of vehicle, other consumables used in specimen transport, average (annual) distance traveled, other logistics | Fuel Price General market research, Accounting office |
|  | Other admin costs |  | Accounting office |
